# Curricular evaluation of “SHOKUIKU program” as a postgraduate minor course of food and nutrition education using a text-mining procedure

**DOI:** 10.1186/s40795-018-0246-7

**Published:** 2018-11-22

**Authors:** Tomoko Ishikawa, Yoko Sato, Kyoko Kurimoto, Yasuko Sone, Rie Akamatsu, Yoko Fujiwara

**Affiliations:** 1 Institute for Human Life Innovation, Ochanomizu University, 2-1-1 Otsuka, Bunkyo-ku, Tokyo, 112-8610 Japan; 2 Natural Science Division, Faculty of Core Research, Ochanomizu University, Tokyo, 112-8610 Japan; 3 Faculty of Health & Nutrition, Takasaki University of Health and Welfare, 37-1 Nakaorui-machi, Takasaki-shi, Gunma 370-0033 Japan

**Keywords:** Diet, food, and nutrition, Training programs, Program appropriateness, Text mining, Qualitative evaluation

## Abstract

**Background:**

**“**SHOKUIKU”, food and nutrition education, is a national promotion to enable people to acquire an adequate knowledge of SHOKU (which include food, nutrition, dietary habits, etc.) in Japanese society and to allow people to make appropriate SHOKU choices in Japan. In order to educate SHOKUIKU experts who can promote evidence-based SHOKUIKU with advanced professional knowledge and skills, an original “SHOKUIKU program” was established. To evaluate this program, a short answer questionnaire was given to students. Results were objectively analyzed by text mining procedures.

**Methods:**

Five hundred forty four comment papers submitted by a total of 52 consenting students after each lecture in the 12 omnibus-style lectures were examined as cross-sectional data. A total of 2507 sentences were decomposed into words, and word classes of morpheme in Japanese were properly specified. Subsequently, on the basis of a constructed keyword data base, 123 morphemes with high frequency were investigated with co-occurrence network analysis. Furthermore, multivariate network analyses according to the student’s major were performed.

**Results:**

Students majoring in food and nutritional sciences recognized that evidence-based SHOKUIKU is “difficult” but “necessary” to “convey” reliable information at “actual” SHOKUIKU sites. On the other hand, students studying other majors not only got an “interesting” opportunity to “learn” “nutrition” and “eating habits” but also thought about their own SHOKUIKU promotion in relation to their major.

**Conclusions:**

These results suggest that the students of the Food Course assumed that they would practice the evidence-based SHOKUIKU themselves, while the students of other courses learned new knowledge more passively. The results also confirmed that students comprehensively grasped the 12 omnibus-style lectures and understood the significance of evidence-based SHOKUIKU regardless of their major. Our original educational program could be valuable for postgraduate students to promote SHOKUIKU.

## Background

### Social background

The extension of a healthy life expectancy in Japan has attracted global attention, as Japan has become a super aged society ahead of the world. However, excessive dietary intake and also nutritional deficiency attributed to a poor appreciation for diet and nutrition remains, even in developed countries [1, 2]. Japan is no exception, and it is imperative to reduce nutritional disorders including both hypernutrition and malnutrition [3].

In 2013, WASHOKU (WA-SHOKU), which means traditional Japanese (WA) food (SHOKU) culture, was chosen for the Intangible Cultural Heritage of the United Nations Educational, Scientific and Cultural Organization (UNESCO) as a social practice based on a set of skills, knowledge, practice, and traditions [4]. As it is known that traditional Japanese food is one of the most healthful and well-balanced diets in the world, the health of people in Japan has benefited from this desirable eating custom. During the 1960s, with international trade liberalization, the traditional Japanese diet based on rice, fish, soybeans, etc. was extended with the addition of meat and dairy products [5]. An intervention study reproducing Japanese diets according to different time periods showed that the Japanese diet in 1975 was the best in nutritional function [6]. However, the original balance of ingredients has gradually collapsed, and lifestyle diseases such as arteriosclerosis, diabetes, and cancer have increased significantly in Japan [7]. To maintain health, it is important to understand the characteristics of the traditional Japanese diet with its significant health benefits, and to practice it.

### Importance of SHOKUIKU

To address such issues and to cultivate desirable food customs in the future, the Japanese Basic Law on SHOKUIKU (SHOKU-IKU), which means food (SHOKU) education (IKU), was established in 2005 [8, 9]. The Basic Law defines SHOKUIKU as acquisition of knowledge about food as well as the ability to make appropriate food choices. To fulfill the principles of the Basic Law, a SHOKUIKU Promotion Plan is formulated every five years in which the key challenges and goals in popularizing the ideas of diet, nutrition, and food culture are established [10]. Though the term SHOKUIKU may sound new, the first appearance of SHOKUIKU dates back to the Meiji era (1868–1912). S. Ishizuka, a military pharmacist, mentioned that physical, intellectual, and moral education is in the end all about SHOKUIKU [11, 12]. His views were incorporated into the Basic Law, in which the Japanese government stated that “SHOKUIKU makes the foundation for living, and is positioned as the base of intellectual (Chiiku), moral (Tokuiku) and physical (Taiiku) education”. In this manner, SHOKUIKU lays the basic foundations for the practice and improvement of life skills. SHOKUIKU includes comprehensive educational activities not only for individuals but also for communities, whereas nutrition education and clinical nutrition instruction are for individuals.

Since the enforcement of the Basic Law, several public and private activities to promote SHOKUIKU have been carried out in various situations, such as at schools, in the local community, and at home. As a result of this wide dissemination of SHOKUIKU, people have begun to show great concern about healthful diets, food safety and conservation of global environments. However, the flood of information, especially uncertain information without scientific evidence, sometimes confuses people. In response to this world wide problem, the American Dietetic Association released a statement urging consumers to consider a meal as a whole, instead of judging specific foods as good or bad [13]. One of the causes of this confusion in Japan is SHOKUIKU professionals’ lack of expertise in nutrition, food culture, production, security, humanities and habits, and practical skills. In Japan, though the training of registered dietitians is carried out in authorized facilities, the training curriculum of SHOKUIKU experts has not been established on similar evidence based foundations. SHOKUIKU must be based on trustworthy evidence from high quality academic research.

### SHOKUIKU curriculum overview

In order to educate SHOKUIKU experts who can promote evidence-based and occasion-suited SHOKUIKU with advanced knowledge based on transdisciplinary viewpoints and practical skills, an original minor course “SHOKUIKU program” was established in 2011 in the Ochanomizu University graduate school, which is a women’s university belonging to the national university corporation [14, 15]. The SHOKUIKU program is composed of five integrated subjects, and the core subject is “Evidence of Dietary Education” which is a multilateral subject consisting of lectures in omnibus form on the importance of evidence in SHOKUIKU, and how to construct and comprehend it.

The most distinctive feature of this program is its integration of arts and sciences. Ochanomizu University graduate school has five major courses (Comparative Studies of Societies and Cultures, Human Developmental Sciences, Gender and Social Sciences, Life Sciences, Advanced Sciences). Any graduate student can register in this program regardless of her major. The purpose of the SHOKUIKU program as a postgraduate curriculum is to expand the curriculum utilizing the character of each academic discipline, and to educate multi skilled and resourceful experts of SHOKU and SHOKUIKU. This feature makes lecture preparation difficult, but it is important to make effective lectures.

There are few universities with curricula specializing in SHOKUIKU education are available. In Italy, undergraduate and graduate programs in Gastronomic sciences in the University of Gastronomic Sciences implemented complementary education in both sciences and humanities, sensory training, and hands-on learning [16]. A practical “SHOKUIKU” program in Japan has been established as an undergraduate minor program in Ehime University to improve students’ own eating behaviors, but not to educate leaders of SHOKUIKU [17]. However, a multidisciplinary and comprehensive academic curriculum in a postgraduate course to educate SHOKUIKU experts is absent except at Ochanomizu University.

### Evaluation of the program

Newly introduced education programs are generally required to objectively validate the educational implications, as has been the case with problem-based learning (PBL) [18, 19]. The SHOKUIKU program is a newly established educational program integrating the arts and sciences, and an evaluation of this program is needed. The aims of this study are to verify whether students of the core subject, “Evidence of Dietary Education” reacted differently to the lectures based on their major, and to see if they were able to gain an integrated understanding of the 12 omnibus-style lectures. To analyze the data obtained, we selected the text-mining method. Text-mining is an exploratory analysis procedure to extract useful information automatically and objectively from huge unstructured textual data. The text-mining tools are being effectively utilized in the interpretation of huge amounts of data in various fields, such as biology, informatics, and education [20, 21]. For the purpose of evaluating the benefits of the lectures, text-mining analyses are also used to interpret comments obtained from students objectively [22, 23]. This study attempts to analyze the answer sentences in a short answer questionnaire obtained from the students registering the core subject “Evidence of Dietary Education”.

## Methods

### Subjects

Sixty graduate students registered in the core omnibus subject of the SHOKUIKU program, “Evidence of Dietary Education”, in 2012 and 2013. From the eligible participants, 52 students consented to this research. The consent rate was 86.7%. Table 1 shows the majors of consenting students. Subjects were divided into the food and nutritional sciences course (Food Course) and otherwise, according to major. The mean attendance rate of the consenting students was 90.5% overall, and there was no difference between the Food Course and otherwise.

**Table 1:** Number of students in major courses analyzed in this study

Major course	Number of students	Groups for comparative analysis
Food and nutritional science	29	Food Course 34
Food and nutritional science (Doctoral program)	5	
Psychology	3	Other Courses 18
Child Studies	1	
Japanese Language and Literature	1	
Comparative Studies of Societies and Cultures (Doctoral program)	1	
Human Environmental Sciences	1	
Biological Sciences	4	
Physics	6	
Chemistry and Biochemistry	1	

Almost all students of the Food Course were graduates of the Department of Nutrition and Food Science, which trains registered dietitians. The students of other courses had not received specialized education about food and nutrition since junior high school or high school. However, the analyses indicated that all students were able to understand the point of the lesson, but there was a difference in recognition of the SHOKUIKU promotion that they will carry out in the future.

### Data collection

The theme of each individual lecture is categorized in Table 2. After each lesson, students were required to submit a comment paper, which was an A5 sized short-answer questionnaire with columns for the date, theme of the lesson, register number, full name, major, grade, and a free space (114 mm × 130 mm) for anything they learned, thought, and felt about the lesson. Comment papers were collected at a total of 24 lessons in 2012 and 2013, and only the papers obtained from consenting students were used. In order to secure anonymity, all textual comments were digitized with the theme of the lesson and the major of the individual. The digitizing data were checked by multiple researchers.

**Table 2:** Categorization of subject contents

Lesson	Theme
1	Introduction
2	Cookery science
3	Japanese food culture
4	Food chemistry
5	Functional food
6	Nutritional chemistry
7	Biological function
8	Nutritional epidemiology
9	Promotion of SHOKUIKU
10	SHOKUIKU for handicapped
11	Study for SHOKUIKU
12	SHOKUIKU in a company

### Analytical procedure

Usually in qualitative analyses, content or thematic analysis is conducted. However, the aim of this study is to analyze the co-occurrence of the morphemes (the smallest meaningful unit of language) extracted and categorized from the data, and objectively express their relationships with the majors of the students, and the lecture themes, respectively. For this, we chose the text mining method to attempt to show the combinations of morphemes that were reported by the respondents. The quantitative text mining analyses [24, 25] were performed using a free software, KH Coder [26, 27], which can analyze Japanese, English, German, Italian, Portuguese and Spanish text. In this study, ChaSen [28], a Japanese morphological analysis tool retained by Matsumoto Laboratory at Nara Institute of Science and Technology was used as the backend program. KH Coder provides a morpheme frequency table and visualizes co-occurrence networks among morphemes using the R [29], which is an integrated suite of software functions for statistical computation and graphical display provided by GNU projects [30].

The data consisting of 546 comments were served for sequential quantitative analysis. All data were automatically decomposed into morphemes, then extracted morphemes were classified into parts of speech, the appearance frequencies were calculated, and the relationships between each morpheme were analyzed. Next, the morphemes that were categorized into compound or unknown terms were checked and confirmed with the original comment sentence retroactively in order to pick up specific compound terms and technical terminology concerning this program such as cookery science and registered dietitian. Finally, the interrelationships among morphemes were visualized as the co-occurrence networks.

All original sentences were analyzed in Japanese, and the final results were translated into English for this report.

## Results

### Morpheme analysis

Using the automatic morpheme analysis, 4044 terms were extracted from all comments, classified into 13 parts of speech, the appearance frequencies were calculated, and the relationships between each morpheme were analyzed (Table 3). The common terms reported by the participants were “research (245)”, “SHOKUIKU (237)”, and “evidence (219)”.

**Table 3:** Appearance frequency of extracted morphemes

noun		sahen noun		na-adjective		organization name		place name		nai adjective		adverb/noun		force extraction term		interjections		verb		adjective		adverb		noun C	
self	146	research	245	necessary	104	Ajinomoto	13	Japan	83	problem	41	now	93	SHOKUIKU	237	thanks (hiragana)	78	think	747	significant	138	again	68	SHOKU	196
foodstuff	106	topic	132	various	97	Kirin	5	China	31	difference	18	today	78	evidence	219	to	13	feel	333	numerous	129	actual	61	person	169
information	100	talk	126	very	78	McDonald’s	3	America	19	means	12	this time	50	interest	199	na	13	know	214	difficult	77	first	55	taste	52
knowledge	95	cooking	111	important	75	S&B Foods	3	American	15	difference (hiragana)	3	usual	48	scientific	104	o	9	consider	212	pleasant	69	specially	53	body	46
teacher	78	understanding	103	request	64	Ochanomizu University	1	Ethiopia	10	way	1	a lot of (hiragana)	47	collagen	45	very much	6	listen	141	interesting	65	some	48	mind	43
nutrition	71	lesson	82	health	43	S&B	1	Maldives	7	mistake	1	numerously	44	cookery science	36	sure	5	eat	132	good	62	really	45	eye	33
content	63	explanation	71	question	42	Kikkoman	1	Africa	6			from now on	39	registered dietitian	35	e	3	learn	129	accurate	58	entirely	24	soma	26
field	58	study	57	so	38	Suntory	1	Beijing	5			past	34	attention	34	ma	2	understand	119	big	42	always	17	water	26
viewpoint	52	meal	53	familiar	36	Tamasu	1	Okinawa	4			recently	33	function	27	ah	1	surprised	91	strong	36	why	11	oil	26
language	50	influence	47	precious	31	Disney	1	Japanese	3			result	32	food culture	27	that	1	do	88	detailed	27	not always	7	other	23
effect	49	lecture	46	certain	26	Yakult	1	Australia	2			time	21	importance	26	yeah	1	see	81	high	25	unexpectedly	6	country	19
science	48	sense	42	like	22	health office	1	Saitama	2			future	21	curry powder	22	hey	1	have	74	new	24	variously	6	quantity	18
eating habit	48	cuisine	36	greatly	18	Ministry of Health and Welfare	1	Shanghai	2			present time	19	nutrition science	22	excuse	1	use	60	wide	20	absolutely	6	power	18
component	46	experiment	35	unexpected	17	Kagawa Nutrition University	1	Asia	1			before	17	food and nutritional science	21	nice to meet you	1	convey	54	broad	19	obscurely	5	year	15
reason	40	intake	335	several	17	Tokyo Gas	1	Sydney	1			each	15	cooking method	21	thanks	1	differ	52	few	19	furthermore	5	aspect	15
data	39	story	334	fresh	16	Japan Women’s University	1	New York	1			previously	14	perception	20			make	52	bad	18	most	5	medicine	14
evidence-based	38	image	32	clear	15			France	1			this day	14	consumer	19			say	41	oily	18	simultaneously	5	head	13
Japanese people	38	experience	32	several (hiragana)	13			Brazil	1			daily	13	handicapped	19			get	38	delicious	16	never	4	book	13
company	37	job	32	new	11			Mali	1			all (hiragana)	11	certainty	18			vary	37	joyful	13	likewise	4	distinction	12
stress	34	realization	32	uneasiness	10			Muli	1			all	11	trace	16			concern	36	small	13	vaguely	4	example	12
food (colloquial style)	34	reference	31	complex	10			Europa	1			the first	10	phytochemical	15			investigate	34	deep	12	some degree	3	bottom	9
food	32	activity	30	wonder	9			Asian	1			fact	9	discipline	15			give	31	unconcerned	12	little more	3	hand	9
spice	31	relation	30	diverse	8			Arakawa	1			last week	9	significance	15			work	29	near	12	until now	3	heart	9
environment	30	change	30	various (hiragana)	7			Hong Kong	1			case	8	impressive	14			enjoy	28	great	12	indeed	3	meat	9
chance	27	agreement	29	simple	7			Kochi	1			everyday	8	malnutrition	14			go	28	sweet	10	quite	3	fish	8
liver	26	life style	28	nature	7			Sakado	1			a part	7	active	14			read	28	soft	10	lot of	3	chicken	8
child	26	existence	27	precise	7			Kagoshima	1			the whole	7	possibility	13				27	hard	9	naturally	3	skin	8
world	26	analysis	27	useful	7			Konaka	1			after that	6	Kono teacher	13			arise	27	low	9	apparently	2	salt	7
enzyme	24	education	26	complete	6			Ishikawa	1			recent years	6	children	13			based	25	thick	9	somehow	2	fire	7
specialty	24	statistics	26	weak point	6			Horanai	1			direct	6	myself	13			show	25	young	6	already	2	form	7

### Co-occurrence network analysis

**Fig. 1:**
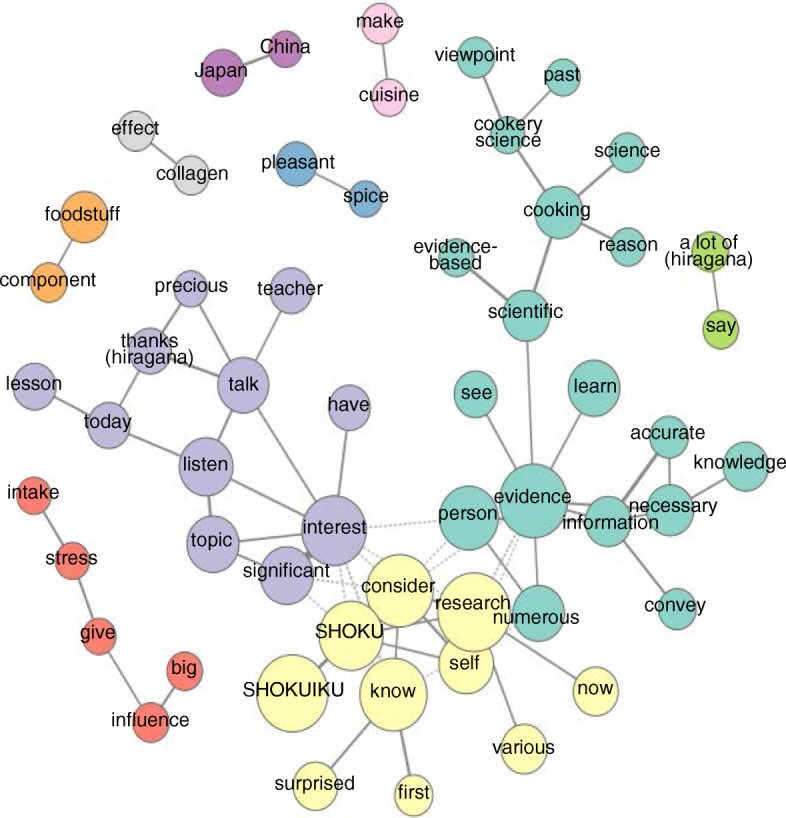
Co-occurrence network among extracted morphemes

One hundred twenty one terms with appearance frequencies between 30 and 300 were served for sequential co-occurrence network analysis. The intensity of co-occurrence relationships among morphemes is indicated in Fig. 1. Including slightly weak relationships, focused morphemes were automatically classified based on centricity in the network. Unlike multidimensional linear measures (MDS), the relationships are significant when two morphemes are joined together in this study. However, if two morphemes are just placed near each other but not bound together, there is not a strong relationship between them. In the figures, a morpheme with higher appearance frequency is indicated as a bigger circle, and automatically classified group is separated by color. Two morphemes that have a strong relationship between them are joined with a bold line.

In Fig. 1, three particularly interesting groups in the morpheme network were revealed. In the first group, the central morpheme was “evidence”, linked to “information-necessary-knowledge”, “accurate”, “learn”, and “scientific” (indicated by moss green in Fig. 1). Students acquired the importance of applying information based on scientific evidence to SHOKUIKU. For this purpose, appropriate knowledge is indispensable. The next group (indicated by yellow in Fig. 1) formed a network with “SHOKUIKU”, “SHOKU”, “know”, “consider”, and “research”. Students knew about various fields of research on food or SHOKUIKU, and considered the relationships between their own research in their major course and SHOKUIKU. In another group (indicated by light purple in Fig. 1), the central morpheme was “interest”, linked to “topic-listen-today-lesson” and “significant”. Students regarded each lesson as a significant and valuable opportunity, and listened with great interest.

### Comparisons between major courses

**Fig. 2:**
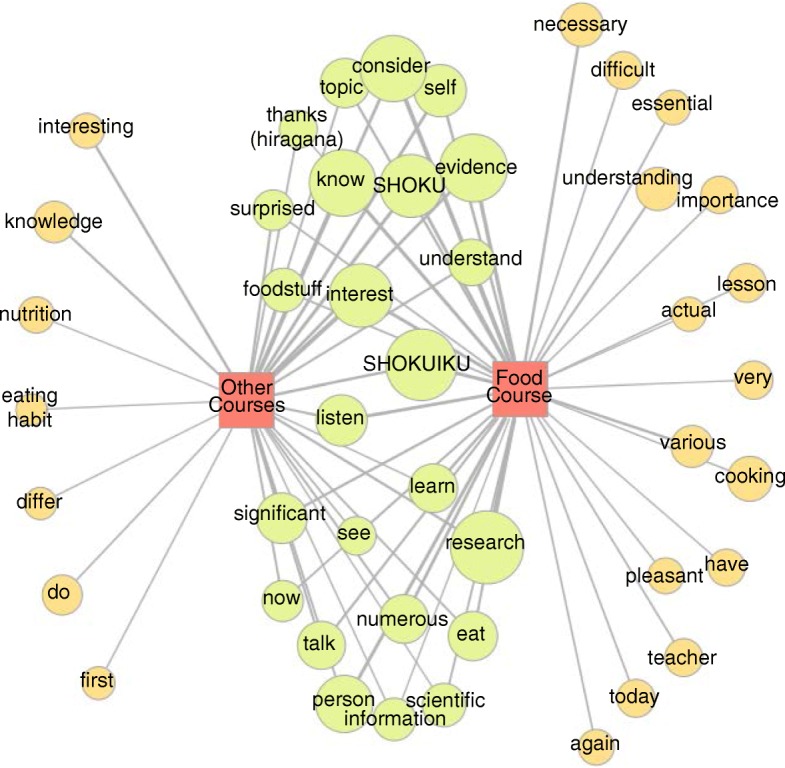
Co-occurrence relationship between major and morpheme

Co-occurrence network analysis methods were used to compare the perceptions of the students in the Food Course with those from other courses. The results are shown in Fig. 2. The students of the Food Course recognized that evidence-based SHOKUIKU was “necessary”, “essential”, and of “importance” at “actual” SHOKUIKU sites, and perceived and referred to it as being “difficult” to “convey” the right information exactly. The students of other courses tended to view the lessons more passively. For example, they mentioned a “first” and “interesting” opportunity to “learn” unknown fields (e.g. “nutrition”, “eating habits”) that “differ” from their major. However, they also referred to situations to improve their own “eating habits” or to act on SHOKUIKU from the viewpoints of their fields.

### Integrated understanding of lesson content

**Fig. 3:**
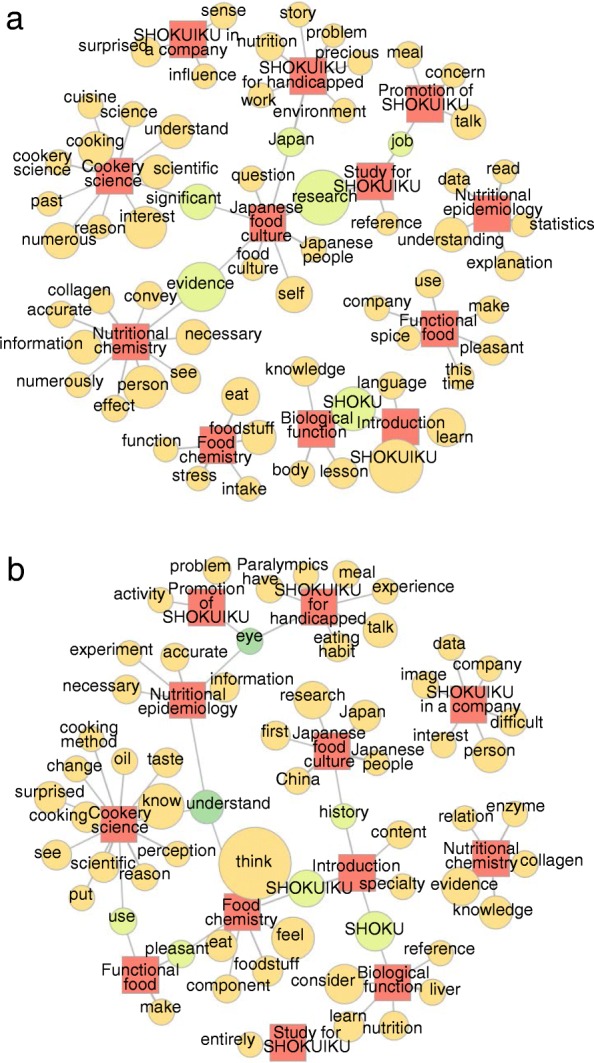
Co-occurrence relationship between theme of lesson and morpheme. **a** Food Course. **b** Other Courses

The integrated understanding of the content of each lesson was then examined in detail (Fig. 3). A total of 126 terms in descending order of the occurrence frequency were used for this analysis for the Food Course and the other courses, respectively.

For Food Course students (Fig. 3a), many morphemes showed that students understood the important points of each lesson, namely “evidence”, “SHOKU”, “SHOKUIKU”, “interest” and “person”. Moreover, the verb “think”, which implies a simple impression, was associated with few comments. Strong co-occurrence relationships between a term and the theme of each lesson were found as follows; “science” correlated to the theme “Cookery science”; “evidence” or “person” correlated to the theme “Nutritional chemistry”; and “SHOKU” correlated to the theme “Biological function”. On the other hand, for students in other courses (Fig. 3b), many of their reports concluded with a verb, such as “think”, “know”, or “feel”. However, the theme of each lesson accurately co-occurred with terms indicating the purpose or point of the lesson. For example, the theme “Nutritional chemistry” correlated to “evidence” and “knowledge”, the theme “Nutritional epidemiology” correlated to “necessary” and “information”, and the theme “Cookery science” correlated to “scientific” and “reason”. The themes strongly co-occurring with “SHOKU” or “SHOKUIKU” were “Introduction”, “Food chemistry”, and “Biological function”.

## Discussion

A core subject, “Evidence of Dietary Education”, of our original postgraduate curriculum training SHOKUIKU experts was evaluated using a text-mining method to analyze short answer questionnaire sentences obtained from students. As a result of the co-occurrence network analysis, we found that the students had different impressions of the lessons according to their major, and that students were able to understand the 12 omnibus-style lectures comprehensively.

In the comparison between the Food Course and the other courses, the difference between the two groups was revealed. The students of the Food Course recognized the importance of accurately communicating correct information, and realized its difficulty and necessity, assuming that they would perform this action themselves at actual SHOKUIKU sites. The students of other courses tended to view the lessons more passively. They were also able to grasp the contents of each lesson clearly, though perhaps it was the initial opportunity to learn about “SHOKU” for most of them. We consider that it is necessary to develop an environment for SHOKU education in junior high school and high school as subjects within home economics or other disciplines and in university as a subject of liberal arts.

From the co-occurrence network analysis with each lesson theme, it became clear that students of both groups comprehensively understood the lecture contents in omnibus form. These results show that regardless of student characteristic, the 12 lecture contents are linked together to cultivate a multifaceted perspective and integrated thinking on SHOKU and SHOKUIKU.

Recently, several competency-based evaluations of education programs have been performed. The development of the competency model in education programs is considered desirable in order to improve the efficiency of the program [31]. In this case, it is possible to evaluate the programs quantitatively and to ask for the achievement of competencies in a pre-code type questionnaire. The desired competencies themselves can then become the evaluation criteria. Using this method, an evaluation survey has been performed for a statewide public health leadership training program [32], and for a peer leader training program in diabetes [33, 34]. The pre-code type questionnaire provides a precise quantitative analysis, but answers are limited by question composition and choices. On the contrary, the short answer questionnaire is useful to penetrate into the subject’s motives. The objective analysis can be performed by text-mining methodology. In the present study, using a quantitative text mining software, KH Coder, multivariate analyses were performed to summarize the data while excluding the intention of researchers, and then co-occurrence networks were visualized to interpret students’ comments.

### Limitations

There are limitations in this study. First is the reliability of automatic analysis by the text-mining software. KH Coder, though it is used worldwide, cannot cover all the technical terms and characteristic compound words by automatic morpheme extraction. To avoid overlooking any morphemes, the original sentence of every extracted morpheme was checked using the concordance function of KH Coder in this study. The second is that the study used cross-sectional textual data obtained from students, so comparison between before and after the lessons was impossible.

## Conclusions

In this study, evaluation of the original minor course for SHOKUIKU in Ochanomizu University graduate school was performed by text mining to analyze the results of short answer questionnaires obtained from students. It was shown that all students comprehensively understood the 12 lecture contents, and achieved beneficial outcomes appropriate to their major as follows. The students of the Food Course recognized the importance of evidence-based SHOKUIKU, and its difficulty. The students of other courses got an opportunity for the first time to learn nutrition, eating habits, and evidence-based SHOKUIKU. The core subject “Evidence of Dietary Education” could be a valuable subject for postgraduate students to take to improve their own awareness and promote SHOKUIKU.

## Abbreviations

MDS: Multidimensional linear measure
SHOKU: Food, nutrition and dietary habits, etc. in Japanese
SHOKUIKU: Food and nutrition education in Japanese
UNESCO: The United Nations Educational, Scientific and Cultural Organization
WASHOKU: Japanese traditional food and cultures

## Authors’ information

TI; Ph. D, project associate professor, in charge of “SHOKUIKU program”.

*fields:* nutrition science, molecular anatomy, medical education, SHOKUIKU.

YSa; Ph. D, assistant professor, registered dietitian, Ochanomizu University SHOKUIKU senior expert. *fields:* cookery science, SHOKUIKU. KK; Ph. D, academic assistant. *fields:* social sciences. YSo; Ph. D, lecturer. *fields:* nutrition science, SHOKUIKU. RA; DrPH., professor, registered dietitian. *fields:* food and nutrition education, SHOKUIKU. YF; Ph. D, professor, project leader of the grant from the Ministry of Education, Culture, Sports, Science and Technology. *fields:* nutrition science, molecular nutrition, home economic education, SHOKUIKU.
